# Metabolomic Analysis of Biochemical Changes in the Plasma of High-Fat Diet and Streptozotocin-Induced Diabetic Rats after Treatment with Isoflavones Extract of Radix Puerariae

**DOI:** 10.1155/2016/4701890

**Published:** 2016-03-03

**Authors:** Yan Zhang, Ping Wang, Youdong Xu, Xianli Meng, Yi Zhang

**Affiliations:** ^1^Department of Pharmacology, School of Pharmacy, Chengdu University of Traditional Chinese Medicine, Chengdu 611137, China; ^2^College of Ethnic Medicine, Chengdu University of Traditional Chinese Medicine, Chengdu 611137, China

## Abstract

The main purpose of this study was to investigate the protective effects of total isoflavones from Radix Puerariae (PTIF) in diabetic rats. Diabetes was induced by a high-fat diet and intraperitoneal injection of low-dose streptozotocin (STZ; 40 mg/kg). At 26 weeks onwards, PTIF 421 mg/kg was administrated to the rats once daily consecutively for 10 weeks. Metabolic profiling changes were analyzed by Ultraperformance Liquid Chromatography-Quadrupole-Exactive Orbitrap-Mass Spectrometry (UPLC-Q-Exactive Orbitrap-MS). The principal component discriminant analysis (PCA-DA), partial least-squares discriminant analysis (PLS-DA), and orthogonal partial least-squares discriminant analysis (OPLS-DA) were used for multivariate analysis. Moreover, free amino acids in serum were determined by high-performance liquid chromatography with fluorescence detector (HPLC-FLD). Additionally, oxidative stress and inflammatory cytokines were evaluated. Eleven potential metabolite biomarkers, which are mainly related to the coagulation, lipid metabolism, and amino acid metabolism, have been identified. PCA-DA scores plots indicated that biochemical changes in diabetic rats were gradually restored to normal after administration of PTIF. Furthermore, the levels of BCAAs, glutamate, arginine, and tyrosine were significantly increased in diabetic rats. Treatment with PTIF could regulate the disturbed amino acid metabolism. Consequently, PTIF has great therapeutic potential in the treatment of DM by improving metabolism disorders and inhibiting oxidative damage.

## 1. Introduction

Diabetes mellitus (DM), a typical complex metabolic disease characterized by insulin deficiency or insulin resistance, can induce a series of complications such as nephropathy, cardiovascular diseases, and diabetic retinopathy. DM was known as “Xiaoke” disease in traditional Chinese medicine. Herbal medications play a particularly important role in the treatment of diabetes for thousands of years and their curative mechanism has been gradually recognized nowadays [1]. For example, isoflavones-containing herbs, also commercially available as dietary supplements, have many reported benefits to cardiovascular and diabetes treatments [2, 3]. Meanwhile, accumulated clinical evidence shows that the conjunction usage of traditional Chinese medicine with western medicine could largely minimize the occurrence of diabetic complications [4].

**Figure 1:**
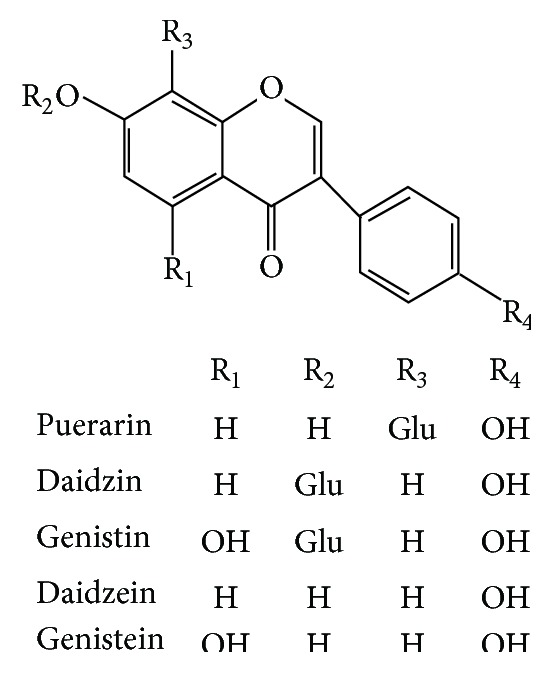
Chemical structure of isoflavonoids from Puerariae Radix.

Puerariae Radix, the dried root of* Pueraria lobata* (Willd.) Ohwi, is also known as Gegen and Kudzu. It is an important source of isoflavones, including puerarin, daidzin, genistin, daidzein, and genistein (Figure 1), which are considered to be the principal bioactive ingredients [5]. Gegen has been used widely in traditional Chinese classical prescriptions for diabetes treatment, such as Gegen Qinlian decoction, Huangqi Gengen decoction, and Yuquan Wan. Emerging evidence also indicates that the extract of Puerariae Radix can improve antioxidant defense system by reducing the plasma level of coenzyme Q9 and MDA [6]. Puerarin has shown great potential benefits in diabetes treatment, not only protecting islets cells from oxidative stress by activating antioxidant enzymes [7], but also greatly improving insulin responsiveness in diabetic mice when used as an adjuvant for metabolic disease [8]. However, the exact therapeutic mechanism of isoflavones extract of Radix Puerariae on diabetes has not yet been well explained.

Metabolomics can provide a systematic overview on the impact of the disease processes and drug interventions, which is well coincident with the holistic theory of Chinese medicine [9]. Recently, metabonomics has been increasingly applied in the diagnosis and evaluation of the therapeutic effects of DM and its complications [10, 11]. For instance, 12 biomarkers were identified using metabolomics in KKay mice, and a water-soluble extract from* Ophiopogon japonicus* has potential activity against diabetes [12]. A recent metabolomic study suggested that 20(S)-ginsenoside Rg3, an active ingredient of* Panax ginseng*, may be involved with the regulation of nucleic acid metabolism, energy metabolism, and gut flora metabolism in type 2 diabetes [13]. Another famous natural product, berberine, might play a pivotal role through downregulating the high level of free fatty acids as the metabolomics study showed for type 2 diabetes treatment [14]. Taken together, these studies unveiled that diabetic diseases have a direct relationship with a disorder in the lipids, fatty acids, and energy metabolism. Additionally, as one kind of metabolites, amino acids especially the branched-chain amino acids (BCAAs) are strongly associated with diabetes and its complications [15] and can presage the development of diabetes [16].

In the development of diabetes and its complications, oxidative stress or imbalance between prooxidants and antioxidants is often thought to be involved [17]. Oxidative stress reflects an imbalance between reactive oxygen species (ROS) and the antioxidants, and high level of this can cause severe cellular dysfunction including membrane lipid peroxidation, DNA fragmentation, and protein damage [18]. On the mechanism of oxidative damage, many studies have identified that the overproduction of superoxide causes the high gene expression of inflammatory cytokines, such as tumor necrosis factor-*α* (TNF-*α*) and vascular endothelial growth factor (VEGF) [19]. In addition, inflammation may cause pancreatic *β*-cell dysfunction, the development of insulin resistance, and so on [20, 21]. In fact, as some experimental and clinical studies have strongly suggested, the treatment based on antioxidant and anti-inflammatory is effective in DM and its complications therapeutic [22, 23].

Considering the DM is associated with metabolic disorders, advanced oxidative stress, and chronic inflammation, we evaluated the therapeutic effects of Puerariae Radix on diabetic rats model induced by a high-fat diet and low-dose streptozotocin and explored the metabolomics profiles using Ultraperformance Liquid Chromatography-Quadrupole-Exactive Orbitrap-Mass Spectrometry and liquid chromatography with fluorescence detector.

## 2. Materials and Methods

### 2.1. Chemicals and Reagents

Puerarin with the batch number of 110752-200912 was bought from NICPBP (China). Daidzin (MUST-15031507), daidzein (MUST-1505-1511), genistin (MUST-14110908), and genistein (MUST-15021802) were bought from the Chengdu Must Bio-Technology Co., Ltd. (Chengdu, China). STZ with the batch number of SLBB7526V were obtained from Sigma Chemical Co. (St. Louis, MO, USA). Water used in this study was prepared by ULUP Ultrapure Water System (Ulupure, Chengdu, China). Methanol and acetonitrile with HPLC-grade were purchased from Thermo Fisher Scientific Inc. (Iowa, USA).

### 2.2. Animals and Experimental Design

Healthy adult male Sprague-Dawley rats weighing (250 ± 20) g were purchased from the Animal Breeding Center of DaShuo Biotechnology Co., Ltd. (Chengdu, China). All rats were maintained on an alternating 12 h light/dark cycle, at a temperature of 22–25°C and humidity of 55%–60%. This study was carried out in China State Administration of TCM, Chengdu University of TCM (TCM Pharmacology P3 laboratory, number TCM2032043). Experimental procedures were strictly in accordance with the Guide for the Care and Use of Laboratory Animals. The animal protocol was approved by the Animal Ethical Committee of Chengdu University of Traditional Chinese Medicine. Rats were divided into normal group and high-fat diet group. Normal group was fed with standard diet. High-fat diet group was fed with high-fat diet for six weeks and then intraperitoneally injected with a single dose of a freshly prepared solution of streptozotocin (40 mg/kg in citric acid-sodium citrate buffer, pH 4.2) [24, 25]. Normal rats received citric acid-sodium citrate buffer only. After STZ injection, all rats were fed with standard diet. Animals with blood glucose levels ≥16.7 mmol/L 72 h after administration of STZ were considered as diabetic rats. Diabetic rats were randomly divided into two groups: the diabetic group and diabetic + PTIF group. At 26 weeks onwards, PTIF was administrated to the treatment rats by intragastric gavage (i.g.) at a dosage of 421 mg/kg. The normal rats and untreated diabetic rats received distilled water the same way. PTIF treatment was performed once daily consecutively for 10 weeks. All animals were allowed to eat and drink freely. At the end of the experiment, plasma/serum samples from each one were isolated by centrifugation at 3500 rpm for 10 min at 4°C. The samples were immediately stored at −20°C until the measurement of oxidative stress and inflammatory cytokines and at −80°C until the metabolomics and amino acid analysis were performed.

### 2.3. Preparation of PTIF

PTIF was provided by Taiji Group, Chongqing Fuling Pharmaceutical Factory Co., Ltd. (China). Isoflavones extract of Radix Puerariae:* Pueraria* powder was extracted with 10 times or 8 times volume of 60% ethanol for 1 h, respectively. The solvent was recovered under reduced pressure. Macroporous resin was used for the purification of the concentrated extraction solution. Isoflavones extract of Radix Puerariae was obtained with vacuum concentration, decompression drying and finally through a 100-mesh sieve.

### 2.4. Standardization of PTIF

A high-performance liquid chromatography method was established for the identification of the major compounds in PTIF (Agilent 1260 HPLC system, Agilent, CA, USA). A Capcell PAK C-18 analytical column (100 mm × 2.0 mm, 3 *μ*m, Shisedo, Japan) was used with the column temperature maintained at 35°C. 0.1% formic acid in water and 0.1% formic acid in acetonitrile were treated as mobile phases A and B, respectively. The mobile phase gradient elution was programmed as follows: 90% A (0.01−12 min), 90%−80% A (12.1−20 min), and 80%−75% A (20.1−30 min). The flow rate was set at 0.5 mL/min, and the sample injection volume was set at 50 *μ*L.

### 2.5. Measurement of Oxidative Stress and Inflammatory Cytokines

Superoxide dismutase (SOD_1_) levels and hypoxia inducible factor 1 alpha (HIF-1*α*) were measured by a sandwich enzyme immunoassay method using a kit from Uscn Life Science Inc. (Wuhan, China). Plasma Malondialdehyde (MDA) was measured by competitive inhibition enzyme immunoassay technique using a kit from Uscn Life Science Inc. (Wuhan, China). Serum vascular endothelial growth factor (VEGF) and intercellular adhesion molecule -1 (ICAM-1) were measured by a sandwich enzyme immunoassay technique using a kit from R&D Systems, Inc. (USA). The concentration of nitric oxide (NO) in serum was quantified based on the enzymatic conversion of nitrate to nitrite by nitrate reductase using a Nitrotyrosine-EIA kit from R&D Systems, Inc. (USA). All data were carried out by Varioskan Multifunctional full wavelength microplate reader (Thermo Fisher Scientific, USA) according to the manufacturers.

### 2.6. UPLC-Q-Exactive Orbitrap-MS Analysis

The plasm samples were reconstituted to room temperature before analysis. Then, 400 *μ*L acetonitrile was added to 100 *μ*L of sample for protein precipitation. The mixture was vortexed for 3 min and then centrifuged at 12,000 rpm for 10 min at 4°C. Finally, the supernatant was transferred to an autosampler vial for further determination.

All the LC/MS data were acquired using Dionex*™* Chromeleon*™* 6.8 and Thermo Xcalibur software. The plasma samples were performed with a C18 column (Acquity UPLC BEH C18, 2.1 mm × 100 mm, 1.7 *μ*m, Waters, United States). 0.1% formic acid in water and 0.1% formic acid in acetonitrile were considered as mobile phases A and B, respectively. The flow rate of 0.4 mL/min at 35°C was used in linear gradients as follows: 95–90% A (0–3 min), 90–85% A (3–6 min), 85–60% A (6–20 min), 60–35% A (20–25 min), 35–5% A (25–30 min), and 5% A (30–35 min). The standard positive ion mode was selected under the following conditions: full scan range, 80 to 1200*m*/*z*; scan resolution, 7,000*m*/*z*/s; sheath gas flow rate, 30 arbitrary units; aux gas flow rate, 10 arbitrary units; spray voltage, 3.5 KV; capillary temperature, 350°C; aux gas temperature, 200°C. The background ion 445.12503(+) was used as lock mass to ensure the mass calibration accuracy. A sample volume of 5 *μ*L was used for injection.

### 2.7. Identification of Potential Biomarkers

The raw MS data obtained by X calibur were exported to MZ mine 2.14.2 using Thermo MS File Reader program. The main MZ mine parameters were set as follows: mass detector was exact mass at noise level 6.0*E*4 intensity; min time span was 0.1 min with *m*/*z* tolerance 5.0 ppm; chromatogram deconvolution used baseline cut-off algorithm and min peak height with 1.0*E*5; isotopic peaks grouper used lowest *m*/*z* representative isotope and setting maximum charge 1. Each sample data was normalized to total area to correct for the MS response shift prior to multivariate analysis. The information of potential biomarkers was obtained by searching databases of KEGG, Lipidmaps, and Metlin based on the accurate molecular weights and isotope shapes.

### 2.8. Amino Acid Analysis

Free amino acids in serum samples were measured by a HPLC-FLD method using an AccQ·Tag precolumn derivatization technology with waters AccQ·Tag chemistry package (Waters Chemical Co., USA). 70 *μ*L borate extraction agent was added to 10 *μ*L serum, and then 20 *μ*L AccQ-Flour derived reagent was added to the mixture. After incubating at 55°C for 10 min, supernatants were collected for the analysis. The amino acid concentration was measured with high-performance liquid chromatography using an amino acid analysis column (3.9 mm × 150 mm, 4 *μ*m, Waters, USA) and a mobile phase consisting of (A) water containing 10% AccQ·Tag Eluent A and (B) water containing 40% acetonitrile at a flow rate of 1 mL/min at 37°C with an excitation wavelength at 250 nm and an emission wavelength at 395 nm. The mobile phase gradient elution was programmed as follows: 5–98% A (0–0.5 min), 98–93% A (0.5–15 min), 93–90% A (15–19 min), 90–67% A (19–32 min), 67% A (32-33 min), 67–0% A (33-34 min), 0% A (34–37 min), 0–100% A (37-38 min), and 100% A (38–64 min). The sample injection volume was set at 10 *μ*L.

### 2.9. Data Analysis

Data was analyzed using R version 3.1.2 (R Core Team; 2014 R: A Language and Environment for Statistical Computing; R Foundation for Statistical Computing, Vienna, Austria; URL: https://www.r-project.org/.), PCA-DA, PLS-DA, and OPLS-DA in package MUMA (Metabolomics Univariate and Multivariate Analysis, Ver 1.4; Edoardo G, Francesca C, Dimitrios S, Silvia M, Andrea S, and Michela G, 2012). R package version 1.4. https://cran.r-project.org/web/packages/muma/index.html) of R was used for multivariate analysis. All results were presented as the mean ± SD. One-way analysis of variance (ANOVA) was used for significance analysis. Values of *P* < 0.05 were considered statistically significant.

## 3. Results

### 3.1. Standardization of PTIF

**Figure 2:**
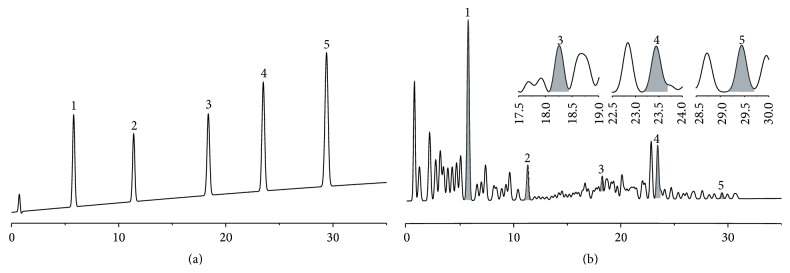
Typical high-performance liquid chromatography profile of five standard mixtures of isoflavonoids (a) and root extract of Puerariae Radix (b) at an absorbance of 254 nm. 1, puerarin (5.8 min); 2, daidzin (11.7 min); 3, genistin (18.2 min); 4, daidzein (23.7 min); 5, genistein (29.3 min).

A typical HPLC fingerprint of PTIF was shown in Figure 2. Five major components were determined by a HPLC method. They were well separated and the retention times are 5.8, 11.7, 18.2, 23.7, and 29.3 min for puerarin, daidzin, genistin, daidzein, and genistein, respectively. As a result, the total isoflavonoids content was 48.00%. As the main constituent, the concentration of puerarin was 19.8%. Daidzin was the next highest at 2.7%, and genistin and daidzein were present at 1.53% and 0.33%, respectively, while genistein has the lowest level at 0.12%.

### 3.2. Effect of PTIF on Body Weight and Blood Glucose Level

As shown in Table 1, compared with the normal group, STZ treatment resulted in a significant decrease in body weight during the 36 weeks of observation. STZ treatment also induced a sustained high blood glucose level as shown in Table 2. While, compared with diabetic group, rats in diabetic + PTIF group showed a significant increase in body weight at weeks 34 and 36 (*P* < 0.05), there was no significant difference in blood glucose with the treatment of PTIP.

### 3.3. Effect of PTIF on Oxidative Stress and Inflammatory Cytokines in STZ-Induced Diabetic Rats

**Figure 3:**
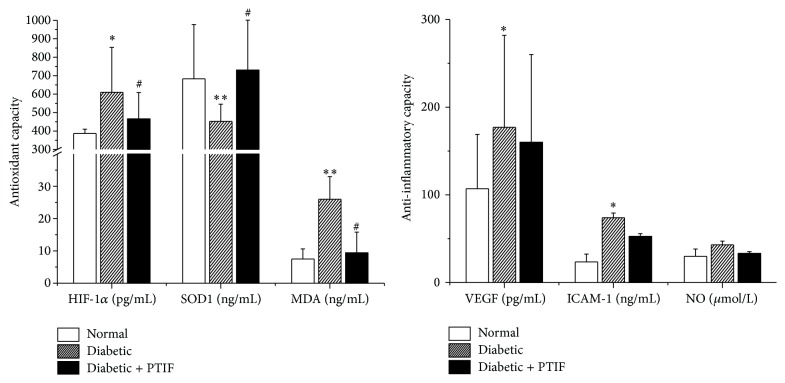
The level of HIF-1*α*, SOD_1_, MDA, VEGF, ICAM-1, and NO in the sample from normal rats, diabetic rats treated with distilled water, and diabetic rats treated with PTIF (421 mg/kg). All data were expressed as mean ± SD. One-way analysis of variance (ANOVA) was used for significance analysis. ^*∗*^ *P* < 0.05 and ^*∗∗*^ *P* < 0.01 indicate a significant difference as compared with normal rats; ^#^ *P* < 0.05, ^##^ *P* < 0.01 indicate a significant difference as compared with diabetic rats.

To investigate the effect of PTIF on antioxidant and anti-inflammatory, we examined the concentration of oxidative stress and inflammatory cytokines. As illustrated in Figure 3, compared with the normal group, the activation of SOD_1_ in diabetic rats was obviously decreased (*P* < 0.01) and significantly increased by PTIF treatment (*P* < 0.05). The concentrations of HIF-*α* and MDA were both remarkably increased and were restored to normal values in PTIF-treated rats. In diabetic rats, we found increased levels of VEGF and ICAM-1 (*P* < 0.05) (Figure 3). Moreover, there were no visible differences in NO levels in the three groups (*P* > 0.05). Treatment with PTIF has no significant influences on inflammatory cytokines, such as VEGF, ICAM-1, and NO in this study (*P* > 0.05).

### 3.4. Validation of UPLC-Q-Exactive Orbitrap-MS Conditions

**Figure 4:**
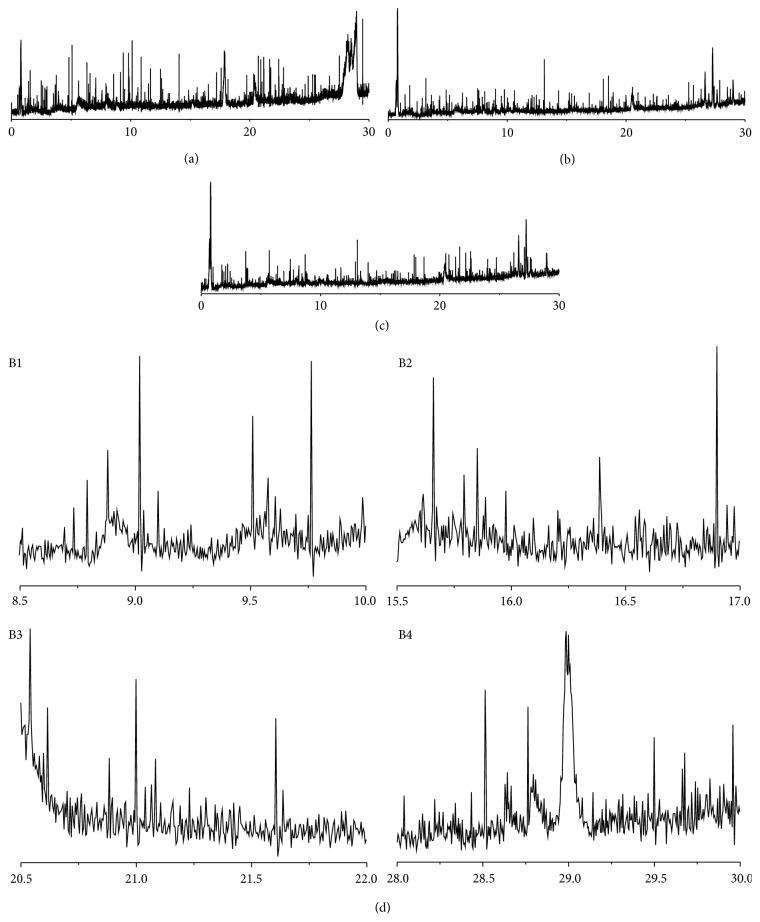
Representative UPLC-Exactive Plus Orbitrap-Mass TIC chromatograms of the plasma samples from the three groups. (a) Normal group, (b) diabetic group, and (c) diabetic + PTIP (421 mg/kg/day) group. (d) B1, B2, B3, and B4 detailed chromatogram B in corresponding time intervals.

The plasma samples were analyzed by UPLC-Q-Exactive Orbitrap-MS according to the method below. Representative UPLC-Exactive Plus Orbitrap-Mass TIC chromatograms of the plasma samples from the three groups are presented in Figure 4. The precision, repeatability of sample preparation, and system stability were validated before the experimental sample analysis. The results indicated that this method could be used in subsequent metabolomics analysis of plasma samples.

### 3.5. Pattern Recognition and Identification of Potential Biomarkers

**Figure 5:**
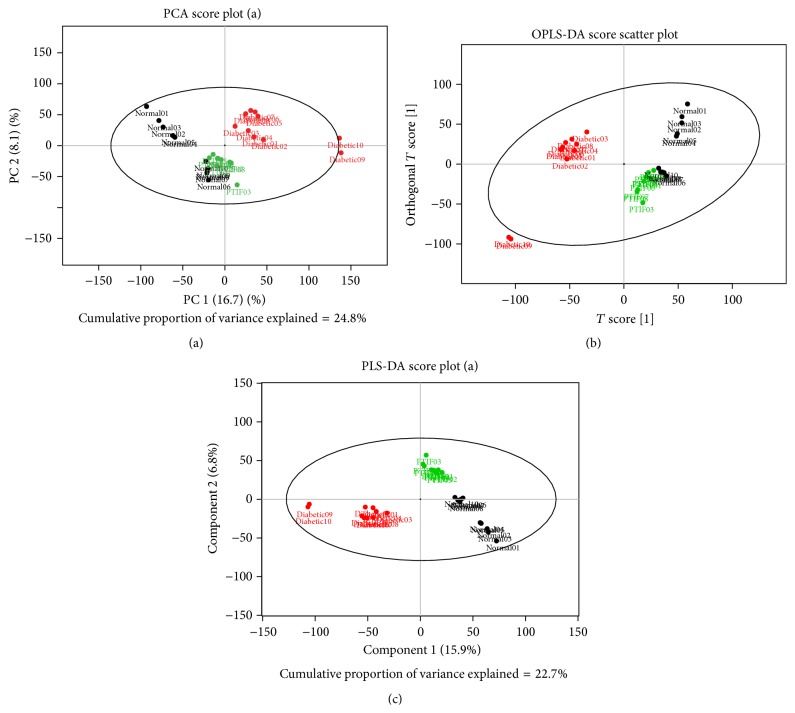
Scores plots from PCA_DA (a), OPLS_DA (b), and PLS_DA (c) classifying normal control group (black dot, *n* = 10), diabetic group (red dot, *n* = 10), and diabetic rats treated with PTIF group (green dot, *n* = 9).

The principal component discriminant analysis (PCA-DA), partial least-squares discriminant analysis (PLS-DA), and orthogonal partial least-squares discriminant analysis (OPLS-DA) are frequently adopted for multivariate analysis. The discovery of potential biomarkers in the plasma enhances our understanding of significant metabolic variations associated with DM rats. As shown in Figure 5, PLS-DA scores plots indicated that there is a satisfactory classification between the normal and diabetic rats, which indicated that the plasma metabolic fingerprint was significantly disturbed by the treatment of streptozotocin. Moreover, biochemical changes of model rats were gradually restored to normal after administration of PTIF, which means that PTIF have a significant efficacy in the improvement of metabolism disorders in STZ-induced diabetic rats.

**Figure 6:**
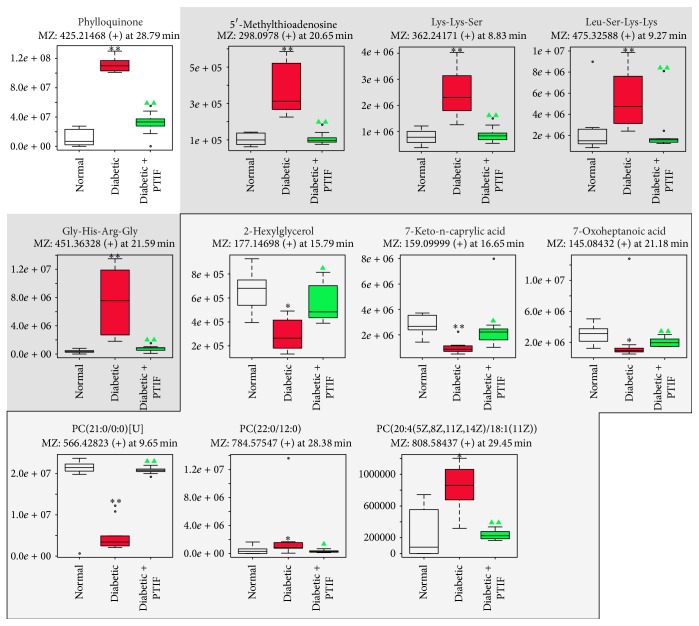
Boxplots of identical biomarkers intensity of rat plasma. For all figures, normal = white bars; diabetic = red bars; diabetic treated with PTIF = green bars; the vertical axis represents the chromatography peak intensity. The biomarkers are possibly related with amino acids, fatty acids, and PCs metabolism. ^*∗*^ *P* < 0.05 and ^*∗∗*^ *P* < 0.01 indicate a significant difference as compared with normal rats; ▲  *P* < 0.05, ▲▲  *P* < 0.01 indicate a significant difference as compared with diabetic rats.

Furthermore, eleven potential metabolite biomarkers were identified and their functional pathways have been analyzed (shown in Table 3). Boxplots of identical biomarkers intensity of rat plasma were shown in Figure 6. In particular, the lipid metabolism-related metabolites, including PC(21:0/0:0)[U], 2-hexylglycerol, 7-keto-n-caprylic acid, 7-oxoheptanoic acid, PC(22:0/12:0), and PC(20:4(5Z,8Z,11Z,14Z)/18:1(11Z)), have been identified in diabetic rats, indicating a dysregulation of lipid metabolites in diabetic rats. Compared with normal control group, the phylloquinone level was significantly increased in diabetic model (*P* < 0.01). Moreover, the metabolomic results also illustrated that PTIF possess a therapeutic influence on DM by partially ameliorating the coagulopathy. In addition, 5′-methylthioadenosine, Lys-Lys-Ser, Leu-Ser-Lys-Lys, and Gly-His-Arg-Gly were found to be significantly increased in model rats (*P* < 0.01). As shown in Figure 6, the marker metabolites with dark gray background possibly relate with amino acids metabolism. Treatment with PTIF exhibited a benefit effect on regulating the amino acid metabolism disorders to normal state.

### 3.6. Amino Acid Analysis Conditions

**Figure 7:**
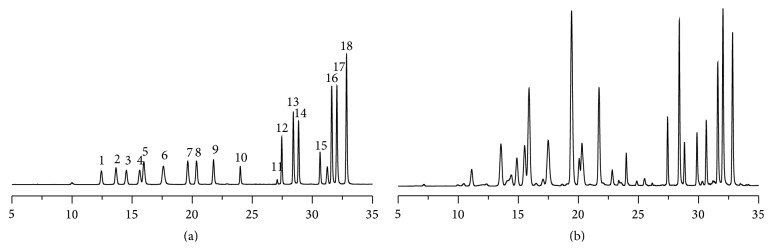
High-performance liquid-fluorescent detection chromatography profile of standard mixtures of amino acids (a) and extract of rat serum (b) by excitation wavelength at 250 nm and the emission wavelength at 395 nm. 1: Asp; 2: Ser; 3: Glu; 4: Gly; 5: His; 6: NH_3_; 7: Arg; 8: Thr; 9: Ala; 10: Pro; 11: Cys; 12: Tyr; 13: Val; 14: Met; 15: Lys; 16: Ile; 17: Leu; 18: Phe.

Free amino acids in serum samples were measured by a HPLC-FLD using an AccQ·Tag precolumn derivatization method. A typical high-performance liquid-fluorescent detection chromatography profile of standard mixtures of amino acids and extract of rat plasma was shown in Figure 7. In the chromatographic conditions established, the calibration curves showed good linearity over the studied concentration range. The within- and between-day variation coefficients were lower than 9.28% and 8.72%, respectively. The spiked recoveries were greater than 75.02%. The results showed that this method was satisfactory for the analysis of amino acids.

### 3.7. Plasma Amino Acid Concentration

Aspartate and cysteine were not detectable in some samples, so the statistical analyses of aspartate and cysteine were not involved. As shown in Table 4, compared with normal control group, the concentrations of the BCAAs (leucine, valine, and isoleucine) were significantly increased in diabetic rats (*P* < 0.01). Furthermore, the levels of glutamate, arginine, and tyrosine in serum were also significantly increased (*P* < 0.01). PTIF supplementation in diabetic rats can lower the level of glutamate and leucine (*P* < 0.05).

## 4. Discussion

It is known that the traditional Chinese medicine therapy was focused on the system function of the body. Therefore, there are many advantages in the treatment of metabolic diseases like diabetes. For the concern about the hypoglycemic effect, the majority of diabetic patients commonly choose a combination therapy of certain antidiabetic drugs and adjuvants such as traditional Chinese medicine, instead of taking antidiabetic drugs alone. The present study shows that PTIF have no significant effect in restoring the disordered blood glucose in DM rats. Thus, it suggests that the therapeutic effect of PTIF might be achieved without improvement of the hyperglycemia of high-fat diet and STZ-induced diabetic rats.

Studies have pointed out that the abnormal metabolic and hyperglycemia-induced oxidative stress leads to a substantial increase in superoxide production, which can activate the protein kinase C, polyol, and hexosamine pathway, thus increasing the conformation of the advanced glycation end products (AGEs) and the expression of the AGEs receptor [19]. Studies have shown that, among the oxidative stress markers, SOD values and lipid peroxidation products such as MDA were highly correlative in diabetic patients, possibly causing further damage to metabolic systems [26]. Some previous research suggests that the beneficial effect of PTIF might result from its intervention in superoxide production. In our research, an obvious decrease in the level of SOD_1_ and a concomitant increase in the concentration of MDA and HIF-1*α* were observed in diabetic rats. This is in agreement with those reported earlier suggesting that the oxidative stress is one of the important events in diabetes. Further, after the administration of PTIF, the plasma levels of MDA, HIF-1*α*, and SOD_1_ were restored, indicating the possible mechanism of PTIF on DM may be correlated with the inhibition of chronic oxidative damage.

Inflammation is a multicomponent response to tissue stress, injury, and infection, which is considered to be the foreground of the acute and chronic pathological conditions [27]. It is now increasingly appreciated that diabetes is a typical disease associated with such chronic inflammatory changes [28]. It is recognized that the low-term chronic inflammation increases insulin resistance leading to hyperglycemia [29]. Various studies have demonstrated that the levels of inflammatory cytokines, including ICAM-1, TNF-*α*, and IL-6, are increased in patients and in diabetic rats [30, 31]. In the present study, a significantly increased concentration of ICAM-1 and VEGF in the diabetic rats probably suggests that the inflammatory processes may contribute to the development of diabetes mellitus. However, we did not see obvious effect of PTIF on VEGF, ICAM-1, and NO, implying that the antidiabetic effect of PTIF may be unrelated to lowering inflammatory cytokines.

In this study, some lipid metabolism-related metabolites, including PC(21:0/0:0)[U], 2-hexylglycerol, 7-keto-n-caprylic acid, 7-oxoheptanoic acid, PC(22:0/12:0), PC(20:4(5Z,8Z,11Z,14Z)/18:1(11Z)), have been identified in diabetic rats, which indicate a dysregulation of lipid metabolites in diabetic rats. Metabolites of the phospholipids and fatty acid pathway are associated with diabetes. Phosphatidylcholines (PCs) products or metabolites are the important components of lipid bilayer of cells, as well as being involved in metabolism and signaling. Previous studies have shown that Radix Puerariae flavones can improve lipid metabolism in adipose tissue and liver in ovariectomized rats due to its estrogen-like effect [32]. Similar to previous studies, this study also shows that the administration of PTIF may contribute to improving the lipid metabolism in diabetic organism. An earlier study suggested that the peroxisome-proliferator activated receptors (PPAR), involved in lipid homeostasis and metabolism, were activated by isoflavones (genistein or daidzein) [33]. Thus, we can conclude that the improvement of PTIF on lipid metabolism may be due to these active ingredients.

As a purified form of vitamin K, phylloquinone was found to play an essential role in the coagulation process. It was widely used for the treatment of disease characterized by reduced levels of prothrombin. It has been shown that endothelial dysfunction, coagulative activation, and platelet hyperreactivity contribute to the hypercoagulable and prothrombotic state in diabetes mellitus, resulting from the interaction among hyperglycemia, insulin resistance, inflammation, and oxidative stress [34, 35]. Moreover, the high blood viscosity, tortuous microvessels, and large platelets induced by diabetes are an important factor in thrombosis in microvessels [36]. Compared with normal control group, the phylloquinone level was significantly increased in diabetic model. Moreover, the metabolomic results also illustrated that PTIF possess a therapeutic effect on DM through partially regulating the coagulopathy. Previous studies revealed that puerarin, as one of the major isoflavonoids compounds of Gegen, could efficiently lower the erythrocyte aggregation index, RBC aggregation, plasma viscosity, and blood yield stress in the acute blood-stasis model rats [37, 38].

Additionally, some potential biomarkers related to amino acid metabolism, including 5′-methylthioadenosine, Lys-Lys-Ser, Leu-Ser-Lys-Lys, and Gly-His-Arg-Gly, have been identified, which suggest an occurrence of amino acid metabolism disorder in diabetic rats. In addition to being an important energy source, amino acids (AAs), especially branched-chain amino acids, (BCAAs) are important intracellular signal molecules that regulate gene transcription and translation [39]. Adenine and yield 5-methylthioribose-1-phosphate are two main metabolites of 5′-methylthioadenosine (MTA). The metabolism of MTA plays a key role in the purine salvage and methionine pathways [40]. Moreover, it is also reported that increased human urinary MTA is associated with severe combined immunodeficiency syndrome [41]. In our research, compared with normal control group, the concentrations of these biomarkers were found to be significantly increased. However, the increase was significantly reversed by PTIF, which indicated PTIF could distinctly regulate the metabolism of amino acid in DM.

Furthermore, 17 kinds of free amino acid content in serum were measured in order to investigate the effect of PTIF on amino acid metabolism in diabetic rats. Recent studies suggest that higher level of branched-chain amino acid directly contributes to insulin resistance by decreasing the activity of AMP-activated protein kinase and eventually leads to type 2 diabetes [42, 43]. However, on the other hand, the activity of branched-chain alpha-keto acid dehydrogenase complex (BCKDC) is reduced markedly in diabetes rats [44], finally leading to the catabolism barriers and an increasing plasma level of BCAAs. Our results showed that the concentrations of the BCAAs (valine, leucine, and isoleucine), glutamate, arginine, and tyrosine were significantly increased, consistent with the previous reports [45]. The beneficial effects of PTIF on improved amino acid metabolism might be explained by its benefit effect on circulating insulin concentrations and insulin responsiveness by activating the signaling pathway of cAMP/PKA-dependent ERK1/2 [46, 47].

Glutamate was a central junction in both amino acid synthesis and degradation [48]. Studies have shown that in pancreatic *β*-cells glutamic acid oxidation and the glutamine-to-glutamate ratio are closely related to the insulin resistance [49]. Additionally, the glutamate level was found to be significantly increased in obese syndrome animals [50], which is similar to the results of our study. Our data also indicate that concentration of glutamate was significantly decreased with the oral administration of PTIF. Arginine is the precursor of nitric oxide (NO) and insulin can stimulate NO(x) synthesis from arginine [51]. Therefore, we can conclude that the increased level of arginine in this study may cause further damage on blood vessels and cell function.

## 5. Conclusion

In summary, a metabolomics method based on UPLC-Q-Exactive Orbitrap-MS has been established to investigate the metabonomic profiles of high-fat diet and STZ-induced diabetic rat. Eleven potential metabolite biomarkers in plasma were identified. The protective effect of PTIF has been reliably confirmed by intervening in lipid metabolism, amino acid metabolism, and coagulopathy. Moreover, we considered that oxidative stress and inflammatory reactions play a key role in diabetic rats. PTIF has great therapeutic potential in the treatment of diabetes mellitus by inhibition of oxidative damage.

## Figures and Tables

**Table 1:** Effects of PTIF (421 mg/kg) on body weight in high-fat diet and STZ-induced diabetic rats (g).

Group	*N*	0 weeks	26 weeks	30 weeks	34 weeks	36 weeks
Normal	10	283.4 ± 7.8	522.2 ± 22.0	553.6 ± 24.4	586.2 ± 30.4	601.8 ± 30.5
Diabetic	10	280.1 ± 5.2	404.1 ± 49.6^*∗*^	375.3 ± 50.3^*∗∗*^	378.0 ± 54.8^*∗∗*^	386.4 ± 57.5^*∗∗*^
Diabetic + PTIF	9	285.3 ± 7.3	426.8 ± 39.5	406.8 ± 48.1	431.7 ± 48.0^#^	438.9 ± 49.4^#^

All data were expressed as mean ± SD. One-way analysis of variance (ANOVA) was used for significance analysis. ^*∗*^ *P* < 0.05 and ^*∗∗*^ *P* < 0.01 indicate a significant difference as compared with normal rats; ^#^ *P* < 0.05 indicate a significant difference as compared with diabetic rats.

**Table 2:** Effects of PTIF (421 mg/kg) on blood glucose in high-fat diet and STZ-induced diabetic rats (mmol/L).

Group	*N*	0 weeks	26 weeks	30 weeks	34 weeks	36 weeks
Normal	10	6.4 ± 0.5	6.8 ± 0.7	6.2 ± 0.5	6.4 ± 0.4	6.6 ± 0.6
Diabetic	10	6.5 ± 0.7	25.1 ± 2.9^*∗∗*^	25.0 ± 3.5^*∗∗*^	23.9 ± 4.0^*∗∗*^	25.1 ± 3.4^*∗∗*^
Diabetic + PTIF	9	6.2 ± 0.6	25.0 ± 2.1	23.6 ± 1.9	23.5 ± 3.0	22.5 ± 3.3

All data were expressed as mean ± SD. One-way analysis of variance (ANOVA) was used for significance analysis. ^*∗∗*^ *P* < 0.01 indicate a significant difference as compared with normal rats.

**Table 3:** Identification results of main potential biomarkers changes.

Number	RT (min)	Formula	Mass (*m*/*z*)	Potential biomarkers	Diabetic versus Normal	Treatment versus Diabetic
1	8.83	C_15_H_31_N_5_O_5_	362.24	Lys-Lys-Ser	↑^*∗∗*^	↓^##^
2	9.27	C_21_H_42_N_6_O_6_	475.33	Leu-Ser-Lys-Lys	↑^*∗∗*^	↓^##^
3	9.65	C_29_H_60_NO_7_P	566.43	PC(21:0/0:0)[U]	↓^*∗∗*^	↑^##^
4	15.79	C_9_H_20_O_3_	177.15	2-Hexylglycerol	↓^*∗*^	↑^#^
5	16.65	C_8_H_14_O_3_	159.1	7-Keto-n-caprylic acid	↓^*∗∗*^	↑^#^
6	20.65	C_11_H_15_N_5_O_3_S	298.1	5′-Methylthioadenosine	↑^*∗∗*^	↓^##^
7	21.18	C_7_H_12_O_3_	145.08	7-Oxoheptanoic acid	↓^*∗*^	↑^##^
8	21.59	C_31_H_46_O_2_	451.36	Phylloquinone	↑^*∗∗*^	↓^##^
9	28.38	C_42_H_84_NO_8_P	784.58	PC(22:0/12:0)	↑^*∗*^	↓^#^
10	28.79	C_16_H_27_N_9_O_5_	425.21	Gly-His-Arg-Gly	↑^*∗*^	↓^##^
11	29.45	C_46_H_82_NO_8_P	808.58	PC(20:4(5Z,8Z,11Z,14Z)/18:1(11Z))	↑^*∗*^	↓^##^

“↑” and “↓” represent upregulation and downregulation. ^*∗*^ *P* < 0.05 and ^*∗∗*^ *P* < 0.01 indicate a significant difference as compared with normal rats; ^#^ *P* < 0.05, ^##^ *P* < 0.01 indicate a significant difference as compared with diabetic rats.

**Table 4:** Effects of PTIF (421 mg/kg) on amino acids in high-fat diet and STZ-induced diabetic rats (*μ*mol/L).

Amino acid	Normal	Diabetic	Diabetic + PTIF
Serine	103.1 ± 30.4	117.7 ± 35.8	115.3 ± 18.2
Glutamate	29.3 ± 7.3	61.0 ± 22.3^*∗∗*^	42.9 ± 8.1^#^
Glycine	111.4 ± 28.3	111.4 ± 20.4	104.6 ± 17.1
Histidine	158.7 ± 27.0	173.1 ± 24.9	208.6 ± 42.8
Arginine	226.0 ± 56.0	347.9 ± 99.8^*∗∗*^	349.4 ± 59.1
Threonine	89.7 ± 24.1	81.0 ± 18.4	96.3 ± 16.7
Alanine	185.4 ± 37.0	213.2 ± 42.7	171.4 ± 35.6
Proline	84.7 ± 28.0	99.0 ± 34.7	69.1 ± 9.4
Tyrosine	38.0 ± 10.6	59.4 ± 18.9^*∗∗*^	45.9 ± 8.1
Valine	50.6 ± 12.7	107.2 ± 32.2^*∗∗*^	123.0 ± 22.6
Methionine	29.4 ± 9.3	33.5 ± 12.8	28.9 ± 5.2
Lysine	86.0 ± 14.5	70.9 ± 11.1	78.4 ± 17.1
Isoleucine	25.7 ± 6.5	68.5 ± 11.3^*∗∗*^	56.9 ± 14.1
Leucine	42.1 ± 10.7	104.8 ± 17.8^*∗∗*^	84.0 ± 21.9^#^
Phenylalanine	40.5 ± 9.8	52.9 ± 13.6	56.6 ± 10.1

All data were expressed as mean ± SD. One-way analysis of variance (ANOVA) was used for significance analysis. ^*∗∗*^ *P* < 0.01 indicate a significant difference as compared with normal rats; ^#^ *P* < 0.05 indicate a significant difference as compared with diabetic rats.
